# Absolute blood eosinophil count could be a potential biomarker for predicting haemorrhagic transformation after intravenous thrombolysis for acute ischaemic stroke

**DOI:** 10.1186/s12883-019-1359-6

**Published:** 2019-06-13

**Authors:** Neringa Jucevičiūtė, Paulius Mikužis, Renata Balnytė

**Affiliations:** 1 Faculty of Medicine, Medical Academy, Lithuanian University of Health Sciences, Mickeviciaus str. 9, LT-44307 Kaunas, Lithuania; 2 Department of Neurology, Lithuanian University of Health Sciences, Eiveniu Str. 2, LT-50161 Kaunas, Lithuania

**Keywords:** Acute ischaemic stroke, Haemorrhagic transformation, Thrombolysis, Eosinophils, Immune cells, Tissue plasminogen activator

## Abstract

**Background:**

Immune cells are involved in all stages of acute ischaemic stroke (AIS) and possess both neuroprotective and neurodamaging properties. It has been suggested that immune system activation after stroke may be associated with the development of haemorrhagic transformation (HT), which is the main complication limiting the clinical use of intravenous thrombolysis with recombinant tissue plasminogen activator (rtPA) for AIS. The purpose of our study was to analyse the association between absolute eosinophil count (AEC) at admission and the occurrence of HT after intravenous rtPA therapy for AIS**.**

**Methods:**

In this retrospective study we enrolled AIS patients who were treated with rtPA within 4.5 h of symptom onset. Baseline stroke severity was evaluated using the National Institutes of Health Stroke Scale (NIHSS). Patients underwent head computed tomography scans at admission which were repeated 24 h after treatment with rtPA or promptly in case of clinical deterioration. HT was defined as blood at any site in the brain on follow-up head computed tomography scans. Spearman’s rank correlation test was used to analyse the correlation between AEC and NIHSS scores. The optimal AEC cut-off value for predicting HT was calculated using the area under the receiver operating characteristic curve. Multiple logistic regression was used to determine the association between AEC included as a binary variable and the incidence of HT.

**Results:**

The data of 201 patients was analysed (59.7% females; median age 77 years); 23 (11.4%) of them developed HT. The median of AEC was 62.5% greater in the non-HT group compared to the HT group (0.13 ×  10^9^/l and 0.08 × 10^9^/l, respectively, *p* = 0.026). No correlation was found between AEC and baseline NIHSS scores (r = 0.061, *p* = 0.393). AEC ≥ 0.11 × 10^9^/l predicted the occurrence of HT with 69.6% sensitivity and 60.7% specificity. AEC ≥ 0.11 × 10^9^/l was independently associated with a 78% reduction in the odds of developing HT (adjusted odds ratio = 0.223, 95% confidence interval = 0.069–0.723, *p* = 0.012).

**Conclusion:**

Higher values of AEC were associated with lower odds of developing HT, thus, AEC at admission could be considered an independent predictive marker of HT after treatment with rtPA for AIS.

## Background

In 2016 10.2% of total deaths worldwide were attributed to stroke and it was the second leading cause of death, surpassed only by ischaemic heart disease [1]. Up to 87% of all stroke cases are ischaemic [2] and can be treated with intravenous thrombolysis (IVT) using recombinant tissue plasminogen activator (rtPA), currently considered the gold standard treatment for acute ischaemic stroke (AIS). However, the clinical use of IVT is limited due to considerable time restrictions and a significant risk of side effects, most notably haemorrhagic transformation (HT) [3]. HT can occur spontaneously in AIS patients not treated with rtPA [4], however, IVT greatly increases the risk of HT [5].

A number of clinical factors have been shown to be associated with greater risk of HT, including but not limited to older age, greater stroke severity, higher glucose levels, lower glomerular filtration rate (GFR), atrial fibrillation and higher systolic blood pressure [6]. However, further investigations on new risk factors for HT should not cease because a better understanding of the mechanisms involved in the pathogenesis of HT may lead to novel adjunctive therapy approaches for AIS. Recently attention has been drawn to the role of immune cells in the pathophysiology of stroke. AIS is no longer believed to be only a vascular disease, but also an immune-mediated condition [7]. Immune system activation after AIS can have both neuroprotective and neurodamaging effects [8], thus, immune modulation therapies are being assessed as promising novel methods of treatment in AIS [9]. Hypereosinophilia has been reported in literature as an unusual cause of AIS [10, 11], however, the role of eosinophils in AIS without hypereosinophilia is not clear. The aim of this study was to assess whether absolute blood eosinophil count (AEC) at admission is associated with the development of HT after treatment with IVT. This paper presents the results of our study and discusses whether eosinophils could be a potential biomarker for predicting haemorrhagic transformation.

## Methods

The study was conducted in the Hospital of Lithuanian University of Health Sciences Kaunas Clinics. Medical records of AIS patients admitted between January 2014 and April 2018 were retrospectively reviewed.. Patients were included in the study if they received alteplase for AIS and had complete medical records. Patients were excluded if they met any of the following criteria: (1) evidence of acute infection at admission or any infection that occurred during the first 24 h after admission; (2) diagnosed chronic infectious disease; (3) active malignancy, chronic inflammatory disease, autoimmune disease, immunosuppressive drug use; (4) acute myocardial infarction at admission or during the first 48 h after admission; (5) transient ischaemic attack less than 24 h prior to admission; (6) previous stroke with partial recovery; (7) endovascular therapy or carotid endarterectomy following alteplase administration. Out of the 347 patients whose data was collected from medical records, 146 fulfilled one or more of the exclusion criteria, thus, the final sample for data analysis comprised 201 patients.

Medical history, clinical examination findings and 12-lead electrocardiograms were obtained immediately upon hospital arrival. Venous blood samples were collected at admission and the following laboratory values were obtained: complete blood count, serum glucose, creatinine (GFR was calculated using the CKD-EPI formula), electrolyte levels, international normalized ratio (INR) and activated partial thromboplastin time. Diabetes mellitus was defined as previous use of anti-diabetic medication or in case of new diagnosis – according to previously published criteria by the World Health Organisation as having fasting plasma glucose ≥7.0 mmol/l or 2–h plasma glucose ≥11.1 mmol/l [12]. Thrombocytopenia was defined as platelet count < 150, × 10^9^/l at admission.

AIS patients were managed according to a protocol; accordingly, a retrospective study design was feasible. All patients presenting within 4.5 h of symptom onset who were potential candidates for IVT underwent non-contrast head computed tomography (CT) scan and after exclusion of intracranial haemorrhage received treatment with intravenous alteplase. Treatment with IVT was performed in accordance with European Stroke Organisation 2008 guidelines [13]. The total dose of alteplase was 0.9 mg/kg up to a maximum dose of 90 mg: 10% of the total dose was administered intravenously as a bolus, followed by an infusion of the remaining dose over 60 min. Stroke severity was assessed using the National Institutes of Health Stroke Scale (NIHSS) prior to thrombolysis. Patients had their head CT scans repeated 24 h after treatment with IVT or promptly in case of clinical deterioration. HT was defined as blood at any site in the brain on the follow-up head CT scan. Previously published criteria by the ECASS II were used to define symptomatic intracranial haemorrhage (sICH): patients were classified as having sICH if there was blood at any site in the brain on the head CT scan and clinical deterioration or adverse effects suggestive of clinical worsening (e.g., drowsiness, increase in hemiparesis) or if there was ≥ 4 point increase in the NIHSS score [14].

Statistical analyses were performed using SPSS software package version 25.0 (IBM). Descriptive statistics were calculated. Lymphocyte to monocyte ratio (LMR) was calculated by dividing absolute lymphocyte count to absolute neutrophil count; neutrophil to lymphocyte count ratio (NLR) was calculated by diving absolute neutrophil count to absolute lymphocyte count. Histograms and the Shapiro-Wilk test were used to assess data normality; nonparametric tests were used for non-normally distributed variables. The Student’s t-test and the Mann-Whitney U test were used to compare intergroup differences in continuous variables; the chi-square test and the Fisher’s exact test were used to compare intergroup differences in categorical variables. The correlation between continuous variables was analysed using Spearman’s rank correlation. The optimal cut-off value for the ability of AEC to predict HT was calculated using the receiver operating characteristic (ROC) curve. Multiple logistic regression analysis was performed with HT as the dependent variable. Values are given as n (%), mean (standard deviation [SD]), or median (interquartile range [IQR]) unless stated otherwise. All *p* values are two-sided, and p values of less than 0.05 were regarded as statistically significant.

## Results

The study sample included 120 (59.7%) females and 81 (40.3%) males. Their age ranged from 46 to 97 years with a median (IQR) of 77 (70–84). 14 (7.0%) patients developed sICH and 9 (4.5%) had asymptomatic HT. Patients who developed HT were older, had higher INR values and baseline NIHSS scores, lower GFR values, more of them used anticoagulants prior to admission and had atrial fibrillation compared to the non-HT group (*p* < 0.05). No statistically significant difference was found in immune cell counts between the HT and non-HT groups with the exception of AEC which was 62.5% greater in the non-HT group compared to the HT group. AEC in our study group ranged from 0.00 to 0.86, × 10^9^/l with a median (IQR) of 0.12 (0.07–0.20). No difference in immune cell counts was detected when comparing the sICH group to the non-sICH group which included patients who developed asymptomatic HT as well as those who did not develop HT. Baseline characteristics of the study sample based on the development of HT and sICH are shown in Table 1.

**Table 1:** Baseline patient characteristics according to the development of haemorrhagic transformation and symptomatic intracranial haemorrhage

	Non-HT (*n* = 178)	HT (*n* = 23)	*p* value	Non-sICH (*n* = 187)	sICH (*n* = 14)	*p* value
Age, years	76.0 (69.0–83.0)	82.0 (75.0–87.0)	0.020	77.0 (70.0–83.0)	83.0 (75.0–87.3)	0.033
Sex, female	107 (60.1)	13 (56.5)	0.741	111 (59.4)	9 (64.3)	0.717
Time to the start of IVT, min	150.0 (118.0–190.0)	132.0 (110.0–163.0)	0.204	150.0 (118.0–190.0)	122.5 (108.8–160.8)	0.134
Baseline NIHSS	9.0 (6.0–14.0)	14.0 (8.0–18.0)	0.014	9.0 (6.0–14.0)	16.0 (10.3–19.0)	0.021
SBP, mmHg	161.0 (143.0–185.0)	172.0 (156.0–190.0)	0.162	163.0 (143.0–186.0)	167.5 (153.8–188.5)	0.478
DBP, mmHg	90.0 (81.0–100.0)	100.0 (80.0–117.0)	0.193	92.0 (81.0–100.0)	90.0 (80.0–110.5)	0.849
INR	1.01 (0.95–1.07)	1.10 (1.01–1.19)	0.003	1.01 (0.95–1.08)	1.09 (1.02–1.29)	0.073
GFR, ml/min/1.73m^2^	63.51 (50.86–76.19)	56.85 (44.0–63.91)	0.040	62.53 (49.25–74.80)	58.00 (43.47–64.85)	0.256
Blood glucose, mmol/l	6.65 (5.91–7.71)	6.57 (6.02–8.68)	0.736	6.64 (5.91–7.70)	6.56(6.14–8.94)	0.610
Haemoglobin, g/l	134.0 (125.0–143.0)	136.0 (128.0–149.0)	0.399	134.0 (125.0–144.0)	130.5 (124.5–140.5)	0.440
Neutrophils, × 10^9^/l	4.56 (3.63–6.04)	3.97 (3.46–5.53)	0.420	4.57 (3.58–6.06)	3.95 (3.26–4.55)	0.117
Lymphocytes, × 10^9^/l	1.99 (1.46–2.57)	1.73 (1.39–2.73)	0.585	1.98 (1.44–2.57)	1.76 (1.39–2.59)	0.627
Monocytes, × 10^9^/l	0.64 (0.50–0.80)	0.66 (0.52–0.88)	0.480	0.64 (0.50–0.81)	0.69 (0.49–0.92)	0.614
NLR	2.14 (1.57–3.39)	2.27 (1.63–3.50)	0.888	2.19 (1.57–3.44)	2.07 (1.63–2.42)	0.526
LMR	3.01 (2.32–4.57)	2.83 (2.22–3.33)	0.273	3.00 (2.29–4.56)	2.84 (2.39–3.21)	0.425
Eosinophils, × 10^9^/l	0.13 (0.07–0.20)	0.08 (0.05–0.12)	0.026	0.12 (0.07–0.20)	0.09 (0.06–0.21)	0.454
Platelets, × 10^9^/l	217.5 (180.8–252.3)	190.0 (163.0–243.0)	0.097	216.0 (181.0–252.0)	186.0 (150.8–244.8)	0.086
Diabetes mellitus	32 (18.0)	3 (13.0)	0.772	32 (17.1)	3 (21.4)	0.715
Atrial fibrillation at admission	59 (33.1)	16 (69.6)	0.001	66 (35.3)	9 (64.3)	0.031
Previous stroke	21 (19.0)	3 (13.0)	0.742	22 (11.8)	2 (14.3)	0.676
Pre-admission anticoagulant use	14 (7.9)	5 (21.7)	0.049	16 (8.6)	3 (21.4)	0.133
Pre-admission antiaggregant use	33 (18.5)	3 (13.0)	0.773	35 (18.7)	1 (7.1)	0.472

Values are given as n (%), mean (SD), or median (IQR). *DBP* diastolic blood pressure, *GFR* glomerular filtration rate, *HT* haemorrhagic transformation, *INR* international normalized ratio, *IVT* intravenous thrombolysis, *LMR* lymphocyte to monocyte ratio, *NIHSS* National Institutes of Health Stroke Scale, *NLR* neutrophil to lymphocyte ratio, *SBP* systolic blood pressure, *sICH* symptomatic intracranial haemorrhage

**Fig. 1:**
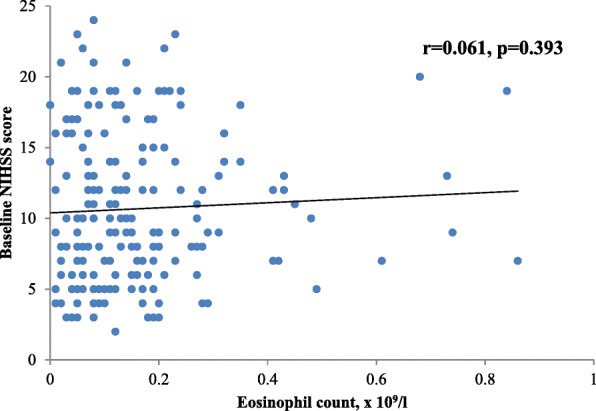
Association between eosinophil count and baseline NIHSS score. *NIHSS = National Institutes of Health Stroke Scale*

Patients with higher AEC tended to have higher baseline NIHSS scores; however, the correlation was weak and statistically insignificant (Fig. 1).

**Fig. 2:**
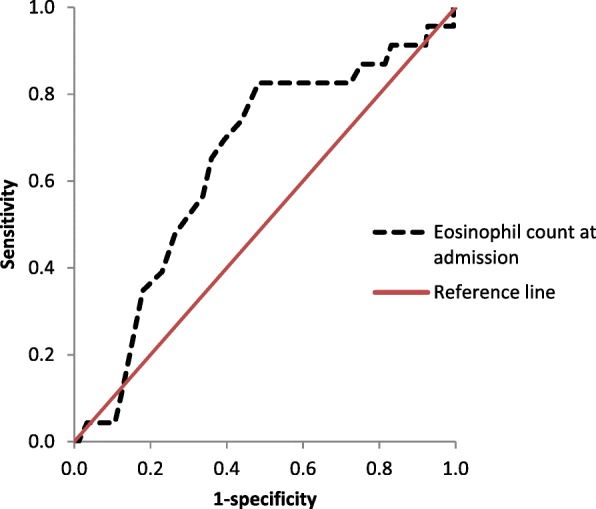
Receiver operator characteristic curves for the prediction of haemorrhagic transformation using eosinophil count

ROC curve analysis showed that the optimal cut-off value for the ability of AEC to predict HT was 0.11 × 10^9^/l with 69.6% sensitivity and 60.7% specificity; area under curve was 0.643 (95% CI = 0.526–0.759, *p* = 0.026) (Fig. 2).

It was also revealed that contrary to HT prediction, AEC is a poor marker for sICH prediction; area under curve was 0.560 (95% CI = 0.400–0.720, *p* = 0.455). The study sample was dichotomized according to AEC: one group was composed of 115 patients whose AEC was at least 0.11 × 10^9^/l and the other group consisted of 86 patients with AEC lower than 0.11 × 10^9^/l. A lower incidence of HT as well as higher platelet, lymphocyte and monocyte counts, higher prevalence of diabetes mellitus and a shorter time interval between symptom onset and the start of treatment with IVT were observed among patients with higher AEC. The characteristics of patients in both groups are summarized in Table 2.

**Table 2:** Baseline patient characteristics dichotomized according to blood eosinophil count

	AEC < 0.11, × 10^9^/l (86, 42.8%)	AEC ≥ 0.11, × 10^9^/l (115, 57.2%)	*p* value
Age, years	79.0 (70.25–84.25)	76.0 (70.0–83.0)	0.530
Sex, female	49 (57.0%)	71 (61.7%)	0.496
HT	16 (18.6%)	7 (6.1%)	0.006
sICH	9 (10.5%)	5 (4.3%)	0.092
Time to the start of IVT, min	160.0 (120.0–196.3)	145.0 (110.0–180.0)	0.048
Baseline NIHSS	9.0 (5.8–16.0)	10.0 (7.0–14.0)	0.416
SBP, mmHg	167.5 (±27.5)	164.7 (±29.6)	0.501
DBP, mmHg	94.7 (±16.4)	92.6 (±17.1)	0.390
INR	1.02 (0.97–1.10)	1.02 (0.95–1.07)	0.214
GFR, ml/min/1.73m^2^	61.23 (44.93–75.52)	62.83 (52.34–73.81)	0.594
Blood glucose, mmol/l	6.59 (5.81–7.72)	6.66 (6.02–7.73)	0.501
Haemoglobin, g/l	134.2 (±15.8)	134.2 (±15.6)	0.996
Neutrophils, × 10^9^/l	4.52 (3.40–6.66)	4.55 (3.65–5.54)	0.434
Lymphocytes, × 10^9^/l	1.61 (1.24–2.16)	2.21 (1.72–2.91)	< 0.001
Monocytes, × 10^9^/l	0.58 (0.46–0.74)	0.68 (0.53–0.85)	0.003
NLR	2.84 (1.75–4.80)	1.91 (1.49–2.91)	0.001
LMR	2.76 (2.06–3.81)	3.20 (2.61–4.82)	< 0.001
Platelets, × 10^9^/l	202.5 (171.0–244.3)	220.0 (183.0–262.0)	0.038
Diabetes mellitus	7 (8.1%)	28 (24.3%)	0.003
Atrial fibrillation at admission	34 (39.5%)	41 (35.7%)	0.573
Previous stroke	8 (9.3%)	16 (13.9%)	0.319
Pre-admission anticoagulant use	10 (11.6%)	9 (7.8%)	0.362
Pre-admission antiaggregant use	16 (18.6%)	20 (17.4%)	0.824

Values are given as n (%), mean (SD), or median (IQR). *AEC* absolute eosinophil count, *DBP* diastolic blood pressure, *GFR* glomerular filtration rate, *HT* haemorrhagic transformation, *INR* international normalized ratio, *IVT* intravenous thrombolysis, *LMR* lymphocyte to monocyte ratio, *NIHSS* National Institutes of Health Stroke Scale, *NLR* neutrophil to lymphocyte ratio, *SBP* systolic blood pressure, *sICH* symptomatic intracranial haemorrhage

**Fig. 3:**
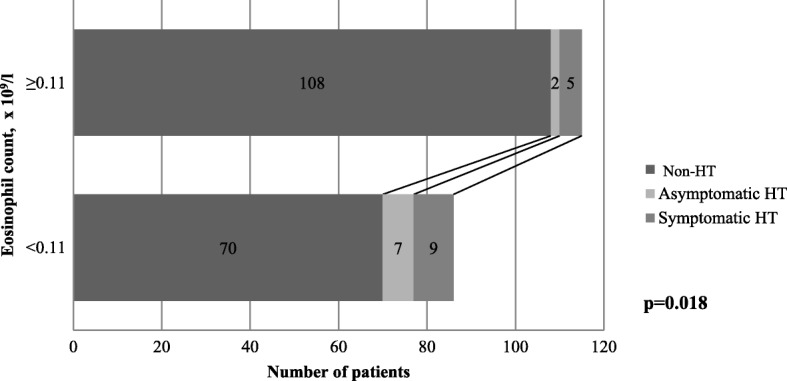
Occurrence of haemorrhagic transformation based on eosinophil count. *HT = haemorrhagic transformation*

2 (1.7%) patients whose AEC was at least 0.11 × 10^9^/l developed asymptomatic HT and 5 (4.3%) developed sICH, whereas the numbers for patients with lower AEC were 7 (8.1%) and 9 (10.5%), respectively, the difference observed between the two groups was statistically significant (*p* = 0.018) (Fig. 3).

**Fig. 4:**
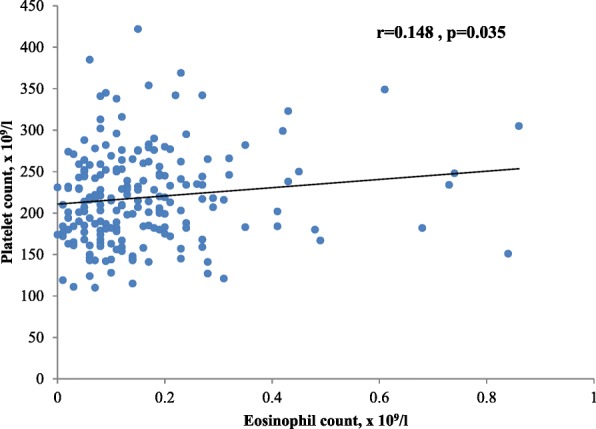
Association between eosinophil count and platelet count

Patients with higher AEC tended to have higher platelet counts; however, the correlation was weak even though statistically significant (Fig. 4). In addition, patients with thrombocytopenia (*n* = 19) and those with platelet count within the normal range (*n* = 182) did not differ in AEC (0.10 and 0.12 × 10^9^/l, respectively, *p* = 0.613).

The results of multiple logistic regression analysis showed that AEC ≥ 0.11 × 10^9^/l was independently associated with a 78% reduction in the odds of developing HT; other variables included in multiple regression analysis were not found to be linked to the occurrence of HT with the exception of baseline NIHSS (Table 3).

**Table 3:** Multiple logistic regression analysis of risk factors for development of haemorrhagic transformation

Variables	Adjusted odds ratio	95% confidence interval	p value
AEC ≥ 0.11, × 10^9^/l	0.223	0.069–0.723	0.012
Baseline NIHSS	1.116	1.005–1.239	0.041

Variables included in analysis are age, time to the start of IVT, baseline NIHSS, INR, GFR, platelet, lymphocyte and monocyte counts, haemoglobin level, dichotomized AEC, diabetes mellitus, atrial fibrillation at admission. All numerical variables were included as continuous variables with the exception of AEC. *AEC* absolute eosinophil count, *GFR* glomerular filtration rate, *INR* international normalized ratio, *IVT* intravenous thrombolysis, *NIHSS* National Institutes of Health Stroke Scale

## Discussion

In this study we discovered that AEC at admission may be a potential prognostic marker for HT development after IVT. We showed that AEC ≥ 0.11 × 10^9^/l was independently associated with a 78% reduction in the odds of developing HT. In addition, AEC was revealed to be a poor marker for sICH development even though patients with sICH tended to have lower AEC values. The differences might have not been detected due to a small sample size and low rate of sICH: out of the 23 patients who developed HT, only 14 had sICH. Few studies have previously looked at the role of eosinophils in AIS: eosinopenia (defined by authors as the percentage of eosinophils < 0.3%) at admission has been reported to be a significant risk factor for mortality at 2 months after AIS [15] and other authors have shown that patients with higher AEC are more likely to have AIS without functional involvement of the limbs compared to patients with lower AEC [16]. Contrary to previously published results by Wang et al. [17], we did not find any significant correlation between AEC and baseline NIHSS. This discrepancy of results may be due to the fact that in the study by Wang et al. [17] all patients presenting with AIS were included independent of the time from symptom onset, whereas we obtained AEC within 4.5 h after the onset of AIS symptoms; it is unknown whether AEC is a dynamic variable as is the case with NLR [18], therefore, results may vary due to possible rapid change of its values after the onset of symptoms.

Neutrophil, lymphocyte and monocyte counts were similar in all groups. NLR also did not differ significantly between groups and these results matched the findings of Guo et al. who reported that NLR values increase after AIS and even though there are no differences in NLR at admission, values measured at least 3–6 h after IVT are greater in patients who later develop parenchymal hematoma or sICH than in those who do not [18]. In our study, LMR values had a tendency to be lower in the HT and sICH groups, however, the differences were not statistically significant. Our results were inconsistent with those reported by Ren et al. who decribed a higher incidence of sICH after IVT in patients whose LMR was < 2.79 at admission [19]. Even though in the study by Ren et al. univariate analysis showed that patients with lower LMR had higher incidence of sICH after IVT, there were additional differences between groups in factors known to be linked to HT: baseline NIHSS, age of patients and prevalence of atrial fibrillation were higher in the group with lower LMR [19], what might explain the difference found in the incidence of sICH by univariate analysis.

When we further compared patients with and without HT, we found a few differences between groups in demographic and clinical factors (e.g., age, GFR values) which were consistent with the results of previous studies [6, 20, 21]. Multiple logistic regression revealed that an increase in baseline NIHSS by one unit leads to a 1.12 fold increase in the odds of developing HT; other factors included in the multiple regression model did not show any significant links to HT with the exception of AEC included as a binary variable.

Early after ischaemia immune cells are released into the bloodstream and infiltrate the brain parenchyma due to increasing blood-brain barrier (BBB) permeability [22, 23]. Out of the several clinical trials which have investigated the efficacy of immunomodulation after AIS, only a few have yielded promising results, among them are interleukin (IL) -1 and lymphocyte-targeted approaches [24]. To our knowledge, eosinophils have not been assessed as a therapeutic target in AIS, probably due to insufficient understanding of the function of eosinophils in cerebral ischaemia. However, there are a few hypotheses which might be explanatory of the association between AEC and HT. Eosinophils are capable of secreting over 35 cytokines, growth factors and chemokines [25], including IL-4 and IL-13 [25–27], which induce the activation of the M2 phenotype microglia [28]. Contrary to the M1 phenotype microglia which contributes to BBB disruption by releasing pro-inflammatory cytokines, proteases and reactive oxygen species, the M2 phenotype possesses neuroprotective properties and may facilitate the resolution of inflammation [28–31]. Another mechanism behind the effect of eosinophils on the attenuated development of HT might be associated with the modulation of angiogenesis. The direct pro-angiogenic effect of eosinophils is mainly attributed to the production of vascular endothelial growth factor (VEGF) [32]. However, VEGF has both neuroprotective and neurodamaging properties, the latter can be attributed to increased vascular permeability [33]. Early intracerebroventricular administration of VEGF decreases infarct volume and reduces ischaemia-induced BBB permeability, contrary to its early intravenous administration, thus, the stimulation of VEGF receptors on luminal and abluminal sites in the brain may lead to the activation of different signalling pathways [34]. If eosinophils are able to extravasate from leptomeningeal vessels and reach abluminal sites in the brain after AIS as is the case with neutrophils [35], then their produced VEGF might be neuroprotective and reduce BBB permeability.. On the other hand, we must emphasize that eosinophils also secrete substances associated with disruptive BBB change, e.g., matrix metalloproteinaise-9 [36] and pro-inflammatory cytokines, such as IL-6 and IL-1 [37–39]. Thus, the effect of eosinophils on the development of HT might depend on the delicate balance between secreted neuroprotective and neurodamaging substances.

Although this study offers new findings on the role of eosinophils in AIS, it also has some limitations. Firstly, this was a retrospective single-centre study with a small sample size, thus, due to the design of the study, there was a higher probability of potential confounding factors. A small sample size might also be explanatory of the wide CI for the adjusted OR of AEC as well as the fact that no association was found between AEC and sICH despite an observed link between AEC and HT. Ergo, it must be emphasized that the precision of our results has limitations and in order to confirm these initial findings, a larger scale multicentre prospective study is necessary. Secondly, we only used baseline values to predict the occurrence of HT even though it is not clear whether AEC is a static variable. Moreover, because we were not able to collect as much information as we intended on patients’ history of allergic diseases, we did not analyse this data and consequently some valuable information might have been missed.

## Conclusions

To sum up, even though our results support the hypothesis that AEC could possibly be considered as an independent predictive marker of HT after AIS treatment with IVT, they are only suggestive of this link. Therefore, larger prospective multicentre studies are needed to further evaluate the temporal dynamics of AEC and its usefulness in predicting the development HT and especially sICH which is the most clinically relevant subtype of HT. A better understanding of the role of eosinophils during stroke is necessary because it could aid in clinical decision making when treating AIS with IVT and lead to novel immunomodulatory therapies for AIS.

## Data Availability

Not applicable.

## Abbreviations

AEC: Absolute eosinophil count
AIS: Acute ischaemic stroke
BBB: Blood-brain barrier
CI: Confidence interval
CT: Computed tomography
DBP: Diastolic blood pressure
GFR: Glomerular filtration rate
HT: Haemorrhagic transformation
IL: Interleukin
IQR: Interquartile range
IVT: Intravenous thrombolysis
LMR: Lymphocyte to monocyte ratio
MMP: Matrix metalloproteinase
NIHSS: National Institutes of Health Stroke Scale
NLR: Neutrophil to lymphocyte ratio
OR: Odds ratio
ROC: Receiver operating characteristic
rtPA: Recombinant tissue plasminogen activator
SBP: Systolic blood pressure
SD: Standard deviation
sICH: Symptomatic intracranial haemorrhage
VEGF: Vascular endothelial growth factor

## References

[1] Global Health Estimates (2016). Deaths by cause, age, sex, by country and by region, 2000-2016.

[2] Alexandru R, Terecoasă EO, Băjenaru OA, Tiu C (2017). Etiologic classification of ischemic stroke: where do we stand?. Clin Neurol Neurosurg.

[3] dela PI, Borlongan C, Shen G, Davis W (2017). Strategies to extend thrombolytic time window for ischemic stroke treatment: an unmet clinical need. J Stroke.

[4] Miller DJ, Simpson JR, Silver B (2011). Safety of thrombolysis in acute ischemic stroke: a review of complications, risk factors, and newer technologies. The Neurohospitalist.

[5] Lees KR, Bluhmki E, von Kummer R, Brott TG, Toni D, Grotta JC (2010). Time to treatment with intravenous alteplase and outcome in stroke: an updated pooled analysis of ECASS, ATLANTIS, NINDS, and EPITHET trials. Lancet.

[6] Whiteley WN, Slot KB, Fernandes P, Sandercock P, Wardlaw J (2012). Risk factors for intracranial hemorrhage in acute ischemic stroke patients treated with recombinant tissue plasminogen activator: a systematic review and meta-analysis of 55 studies. Stroke.

[7] Magnus T, Wiendl H, Kleinschnitz C (2012). Immune mechanisms of stroke. Curr Opin Neurol.

[8] Kamel H, Iadecola C, Implications C, Kamel H, Iadecola C, Implications C (2012). Brain-immune interactions and ischemic stroke: clinical implications. Arch Neurol.

[9] Fu Y, Liu Q, Anrather J, Shi F (2015). Immune interventions in stroke. Nat Rev Neurol.

[10] Khwaja GA, Duggal A, Kulkarni A, Choudhary N, Gupta M, Chowdhury D (2013). Hypereosinophilia-an unusual cause of multiple embolic strokes and multi-organ dysfunction. J Clin Diagnostic Res.

[11] Sethi H, Schmidley J (2010). Cerebral infarcts in the setting of eosinophilia three cases and a discussion. Arch Neurol.

[12] WHO. Definition and Diagnosis of Diabetes Mellitus and Intermediate Hyperglycemia. Who2 2006. doi: ISBN 92 4 159493 4.

[13] Ringleb PA, Bousser M, Ford G, Bath P, Brainin M, Caso V (2008). Guidelines for Management of Ischaemic Stroke and Transient Ischaemic Attack 2008 The European Stroke Organization ( ESO ) Committee and the ESO Writing Committee Executive. Stroke.

[14] Hacke W, Kaste M, Fieschi C, Von Kummer R, Davalos A, Meier D (1998). Randomised double-blind placebo-controlled trial of thrombolytic therapy with intravenous alteplase in acute ischaemic stroke (ECASS II). Lancet.

[15] Hori YS, Kodera S, Sato Y, Shiojiri T (2016). Eosinopenia as a predictive factor of the short-term risk of mortality and infection after acute cerebral infarction. J Stroke Cerebrovasc Dis.

[16] Guo L, Liu S, Zhang F, Mao G, Sun L, Liu Y (2015). The role of eosinophils in stroke: a pilot study. Eur Rev Med Pharmacol Sci.

[17] Wang J, Ma L, Lin T, Li SJ, Chen LL, Wang DZ (2017). The significance of eosinophils in predicting the severity of acute ischemic stroke. Oncotarget.

[18] Guo Z, Yu S, Xiao L, Chen X, Ye R, Zheng P (2016). Dynamic change of neutrophil to lymphocyte ratio and hemorrhagic transformation after thrombolysis in stroke. J Neuroinflammation.

[19] Ren H, Han L, Liu H, Wang L, Liu X, Gao Y (2017). Decreased lymphocyte-to-monocyte ratio predicts poor prognosis of acute ischemic stroke treated with thrombolysis. Med Sci Monit.

[20] Lee J-G, Lee KB, Jang I-M, Roh H, Ahn M-Y, Woo H-Y (2013). Low glomerular filtration rate increases hemorrhagic transformation in acute ischemic stroke. Cerebrovasc Dis.

[21] Prabhakaran S, Rivolta J, Vieira JR, Rincon F, Stillman J, Marshall RS (2010). Symptomatic intracerebral hemorrhage among eligible warfarin-treated patients receiving intravenous tissue plasminogen activator for acute ischemic stroke. Arch Neurol.

[22] Iadecola C, Anrathner J (2012). The immunology of stroke: from mechanism to translation. Nat Med.

[23] Merali Z, Huang K, Mikulis D, Silver F, Kassner A (2017). Evolution of blood-brain-barrier permeability after acute ischemic stroke. PLoS One.

[24] Veltkamp R, Gill D (2016). Clinical trials of immunomodulation in ischemic stroke. Neurotherapeutics.

[25] Davoine F, Lacy P (2014). Eosinophil cytokines, chemokines, and growth factors: emerging roles in immunity. Front Immunol.

[26] Woerly G, Lacy P, Ben Younes A, Roger N, Loiseau S, Moqbel R (2002). Human eosinophils express and release IL-13 following CD28-dependent activation. J Leukoc Biol.

[27] Piehler D, Stenzel W, Grahnert A, Held J, Richter L, Köhler G (2011). Eosinophils contribute to IL-4 production and shape the T-helper cytokine profile and inflammatory response in pulmonary cryptococcosis. Am J Pathol.

[28] Orihuela R, McPherson CA, Harry GJ (2016). Microglial M1/M2 polarization and metabolic states. Br J Pharmacol.

[29] Colton CA, Wilcock DM (2010). Assessing activation states in microglia. CNS Neurol Disord - Drug Targets.

[30] Patel AR, Ritzel R, McCullough LD, Liu F (2013). Microglia and ischemic stroke: a double-edged sword. Int J Physiol Pathophysiol Pharmacol.

[31] Hu X, Li P, Guo Y, Wang H, Leak RK, Chen S (2012). Microglia/macrophage polarization dynamics reveal novel mechanism of injury expansion after focal cerebral ischemia. Stroke.

[32] Puxeddu I, Alian A, Piliponsky AM, Ribatti D, Panet A, Levi-Schaffer F (2005). Human peripheral blood eosinophils induce angiogenesis. Int J Biochem Cell Biol.

[33] Greenberg D, Jin K (2013). Vascular endeothelial growth factros (VEGFs) and stroke. Cell Mol Life Sci Mol Life Sci.

[34] Kaya D, Gürsoy-Özdemir Y, Yemisci M, Tuncer N, Aktan S, Dalkara T (2005). VEGF protects brain against focal ischemia without increasing blood-brain permeability when administered intracerebroventricularly. J Cereb Blood Flow Metab.

[35] Perez-de-Puig I, Miró-Mur F, Ferrer-Ferrer M, Gelpi E, Pedragosa J, Justicia C (2015). Neutrophil recruitment to the brain in mouse and human ischemic stroke. Acta Neuropathol.

[36] Fujisawa T, Kato Y, Terada A, Iguchi K, Kamiya H (1999). Matrix Metalloproteinase-9 in peripheral blood eosinophils. Int Arch Allergy Immunol.

[37] Esnault S, Kelly EAB, Nettenstrom LM, Cook EB, Seroogy CM, Jarjour NN (2012). Human eosinophils release IL-1ß and increase expression of IL-17A in activated CD4 + T lymphocytes. Clin Exp Allergy.

[38] Chu VT, Fröhlich A, Steinhauser G, Scheel T, Roch T, Fillatreau S (2011). Eosinophils are required for the maintenance of plasma cells in the bone marrow. Nat Immunol.

[39] Varatharaj A, Galea I (2017). The blood-brain barrier in systemic inflammation. Brain Behav Immun.

